# Presence of Left Atrial Fibrosis May Contribute to Aberrant Hemodynamics and Increased Risk of Stroke in Atrial Fibrillation Patients

**DOI:** 10.3389/fphys.2021.657452

**Published:** 2021-06-07

**Authors:** Nikhil Paliwal, Rheeda L. Ali, Matteo Salvador, Ryan O’Hara, Rebecca Yu, Usama A. Daimee, Tauseef Akhtar, Pallavi Pandey, David D. Spragg, Hugh Calkins, Natalia A. Trayanova

**Affiliations:** ^1^Alliance for Cardiovascular Diagnostic and Treatment Innovation, Johns Hopkins University, Baltimore, MD, United States; ^2^Department of Biomedical Engineering, Johns Hopkins University, Baltimore, MD, United States; ^3^Department of Mathematics, Politecnico di Milano, Milan, Italy; ^4^Division of Cardiology, Department of Medicine, Johns Hopkins University School of Medicine, Baltimore, MD, United States; ^5^Department of Radiology, Johns Hopkins University School of Medicine, Baltimore, MD, United States; ^6^Department of Medicine, Johns Hopkins University School of Medicine, Baltimore, MD, United States

**Keywords:** atrial fibrillation, stroke, hemodynamics, fibrosis, personalized simulation

## Abstract

Atrial fibrillation (AF) patients are at high risk of stroke, with the left atrial appendage (LAA) found to be the most common site of clot formation. Presence of left atrial (LA) fibrosis has also been associated with higher stroke risk. However, the mechanisms for increased stroke risk in patients with atrial fibrotic remodeling are poorly understood. We sought to explore these mechanisms using fluid dynamic analysis and to test the hypothesis that the presence of LA fibrosis leads to aberrant hemodynamics in the LA, contributing to increased stroke risk in AF patients. We retrospectively collected late-gadolinium-enhanced MRI (LGE-MRI) images of eight AF patients (four persistent and four paroxysmal) and reconstructed their 3D LA surfaces. Personalized computational fluid dynamic simulations were performed, and hemodynamics at the LA wall were quantified by wall shear stress (WSS, friction of blood), oscillatory shear index (OSI, temporal directional change of WSS), endothelial cell activation potential (ECAP, ratio of OSI and WSS), and relative residence time (RRT, residence time of blood near the LA wall). For each case, these hemodynamic metrics were compared between fibrotic and non-fibrotic portions of the wall. Our results showed that WSS was lower, and OSI, ECAP, and RRT was higher in the fibrotic region as compared to the non-fibrotic region, with ECAP (*p* = 0.001) and RRT (*p* = 0.002) having significant differences. Case-wise analysis showed that these differences in hemodynamics were statistically significant for seven cases. Furthermore, patients with higher fibrotic burden were exposed to larger regions of high ECAP, which represents regions of low WSS and high OSI. Consistently, high ECAP in the vicinity of the fibrotic wall suggest that local blood flow was slow and oscillating that represents aberrant hemodynamic conditions, thus enabling prothrombotic conditions for circulating blood. AF patients with high LA fibrotic burden had more prothrombotic regions, providing more sites for potential clot formation, thus increasing their risk of stroke.

## Introduction

Atrial fibrillation (AF) is the most common form of cardiac arrhythmia. About 1–2% of individuals worldwide currently suffer from AF (Andrade et al., 2014), and its prevalence is expected to increase 2.5-fold in the next four decades (Go et al., 2001). Patients afflicted with AF have a higher mortality rate due to dramatically increased stroke risk (Andrade et al., 2014). The left atrium appendage (LAA) has been identified as the most common site for clot formation, with ∼90% clots originating from the LAA causing stroke in AF patients (Kamp et al., 1999; Yaghi et al., 2015). Clinical and computational studies on LAA anatomy and blood flow have established that relatively low blood flow in the LAA explains the propensity to clot formation and eventual stroke events (Lee et al., 2014; Otani et al., 2016; Masci et al., 2019, 2020). In addition to the LAA, increased left atrium (LA) fibrotic burden has been associated with increased risk of stroke in clinical and imaging studies (Daccarett et al., 2011). However, the exact mechanisms by which the presence of fibrosis at the LA wall contributes to increased stroke risk remain unknown. In this study, we aimed to use personalized hemodynamic simulations of LA blood flow to explore the potential mechanisms that explain the increased stroke risk for AF patients with high fibrotic burden in the LA.

Computational fluid dynamic (CFD) analysis on patient-specific image-based LA models is a powerful tool that provides highly resolved physiological blood flow dynamics in the LA, which is not achievable by using current imaging modalities. CFD has been used to investigate how different LAA shapes result in different hemodynamics and to provide insights into their propensity to stroke (Otani et al., 2016; Masci et al., 2019, 2020). Furthermore, CFD studies have explored how LAA closure devices might change the hemodynamics in the LA, which could potentially alter the AF patients’ stroke risk (Paliwal et al., 2020). Here, we use personalized CFD analysis to investigate aberrant hemodynamics in the vicinity of LA fibrotic regions and to dissect the potential mechanisms that lead to increased stroke risk in AF patients with high LA fibrotic burden.

## Materials and Methods

### Patient Image Acquisition and LA Fibrosis Identification

We retrospectively collected imaging data of eight AF patients from the Johns Hopkins Hospital. Preablation late-gadolinium-enhanced magnetic resonance imaging (LGE-MRI) scans were acquired using a 1.5-T Avanto MR system (Siemens Medical Solution, Malvern, PA) for the purpose of visualizing and reconstructing the LA geometry. Scans were performed in the axial orientation 10–27 min following 0.2 mmol/kg of Gadavist (active ingredient, Gadobutrol) agent using a fat-saturated three-dimensional IR-prepared fast spoiled gradient-recalled echo sequence, with ECG-triggered and respiratory navigator gating. For each LGE-MRI, the image resolution was 1.25 × 1.25 × 2.5 mm. Before MRI acquisition, persistent AF patients were either kept on antiarrhythmic medication and/or referred for cardioversion. The MRI was taken during sinus rhythm for paroxysmal AF patients. For each patient, MRI was optimized to perform image acquisition during the diastole of the left atrium.

The geometrical reconstruction workflow of the LA from LGE-MRI is presented in detail in recent publications (Ali et al., 2019; Boyle et al., 2019; Shade et al., 2020). Briefly, the LA epicardial walls were manually delineated on the LGE-MRI images using ITK snap^1^ (Yushkevich et al., 2006). The epicardial segmentation was represented as a triangular surface of connected nodes and edges. An affine transformation was used to ensure alignment of the triangular surface with the LGE-MRI (Cantwell et al., 2014; Ali et al., 2020). The LGE intensity was projected onto the epicardial surface as the maximum voxel intensity through the wall along an inward facing normal (Ali et al., 2020). The image intensity ratio (IIR) was then used to classify surface intensities as fibrotic or non-fibrotic. Fibrosis was identified as intensities that were more than 1.22 times the mean blood pool intensity (Khurram et al., 2014).

### Personalized LA Hemodynamic Modeling

The segmented LA surface was preprocessed to identify the four pulmonary veins (PVs) and mitral valve (MV). The boundaries of all PVs were extended using Meshmixer (Autodesk Research, New York, NY) to represent the entrance length at the inlets for hemodynamic simulations (Karmonik et al., 2008). Volumetric meshing was performed using tetrahedral elements in the LA volume, with three prism layers at the LA wall using ICEM CFD (Ansys, Canonsburg, PA). The average element size was prescribed to be 0.5 mm, resulting in ∼3–8 million elements for each case depending on the size of the LA.

The boundary conditions (BCs) were then prescribed as inlet at the four PVs and zero pressure outlet BC at the MV. To obtain physiological blood flow at the PVs, we interpolated physiological velocity values derived from the literature, and inlet velocities were scaled according to the cross-sectional area of each PV for each model (Gentile et al., 1997; De Marchi et al., 2001). The blood flow was assumed to be in the laminar regime, as flow in the atria has been shown to have Reynolds number < 2,300 (Fung, 2013). Due to high flow in LA vascular structure, blood was modeled as a Newtonian fluid with density and dynamic viscosity values of 1,056 kg/m^3^ and 0.0035 Pa s, respectively. The LA wall was prescribed a predefined motion, and no-slip BC was assumed at the wall. Details of the LA wall motion and inlet velocity from the PVs is provided in the Supplementary Material. The flow-governing Navier–Stokes’ equations were solved to obtain hemodynamics in the LA, as described in our previous publications (Paliwal et al., 2017, 2018). CFD simulations were solved using the open-source OpenFOAM software’s pimpleFoam module^2^. For each case, CFD simulations were performed for three identical cardiac cycles during sinus rhythm to obtain numerical stability, and results from the third cycle were used as final hemodynamic result. The length of the cardiac cycle was equal to 0.92 s, which was discretized with a temporal resolution of 0.001 s using first-order spatial discretization scheme (Paliwal et al., 2017). Hemodynamic results were saved at 92 time intervals for each case, representing the blood flow in the LA with a resolution of 0.01 s. Parallel simulations were performed at the Maryland Advanced Research Computing Center supercomputing facility^3^.

### Hemodynamic Parameters

For each case, simulation results from 92 solution points were exported and used to quantify four hemodynamic metrics defined over a single cardiac cycle in sinus rhythm: time-averaged wall shear stress (WSS), oscillatory shear index (OSI), endothelial cell activation potential (ECAP), and relative residence time (RRT). These parameters are derived from the WSS vector (WSSv), which represents the tangential (along the LA wall) frictional stress of the blood flow at the LA wall and quantifies the imprint of the blood flow in the near vicinity of the LA wall. Time-averaged WSS captures how slow or fast the blood moved at a local point near the LA wall during sinus rhythm. We quantified WSS by averaging the WSSv magnitude over one cardiac cycle in sinus rhythm (Xiang et al., 2011). OSI is a non-dimensional parameter that represents the average directional change in WSSv over the cardiac cycle, capturing the local fluctuations in the blood flow sensed locally at the LA wall. OSI was computed based on the difference in the direction of the WSSv over a cardiac cycle, normalized by its magnitude (Xiang et al., 2011). OSI values can range between 0 and 0.5, with 0.5 representing a 180° change in the WSSv direction during a cardiac cycle. ECAP is the ratio of WSS and OSI, which amplifies the regions of low WSS and high OSI, representing regions of aberrant hemodynamics in vascular flows (Chiu et al., 2003; Himburg et al., 2004; Di Achille et al., 2014). RRT reflects the residence time of flowing blood near the LA wall and is evaluated based on both WSS and OSI. Equations for calculations of these hemodynamic metrices are provided in the Supplementary Material and were calculated using ParaView^4^ (Ahrens et al., 2005). To quantify aberrant flow burden for each LA hemodynamic model, we identified areas at the LA wall that had high ECAP values. Aberrant hemodynamic burden was defined as the area exposed to ECAP above 10% of the average values at the LA wall (Jou et al., 2008; Xiang et al., 2011). For each case, aberrant hemodynamic burden was quantified as the fraction of the total LA surface area that was exposed to high ECAP.

### Registration of Fibrosis on LA Hemodynamic Models and Statistical Analysis

Due to the LA geometry preprocessing, extension of PVs, and LA volume meshing, the LA hemodynamic models did not align with the initially segmented LA geometries. Therefore, to accurately and efficiently register the initial LA segmentation to the final LA hemodynamic models, we employed a projection technique (Regazzoni et al., 2020; Fedele and Quarteroni, 2021). In the framework of the VMTK library (28) (Schroeder et al., 2004), we performed either a finite-element expansion or closest-point interpolation to project imaging data from the initial LA segmentation to the final LA hemodynamic volumetric mesh. Details of the registration method are provided in the Supplementary Material. Briefly, we use a finite-element-based expansion or contraction of the base mesh elements from the initial LA segmentation on the final LA hemodynamic volumetric mesh and then use a closest-point interpolation technique using the VTK library. This registration method assigns to each element of the LA wall in the hemodynamic model an associated LGE-MRI image intensity value from the initial image, the latter used to identify whether that element was fibrotic or non-fibrotic.

**FIGURE 1:**
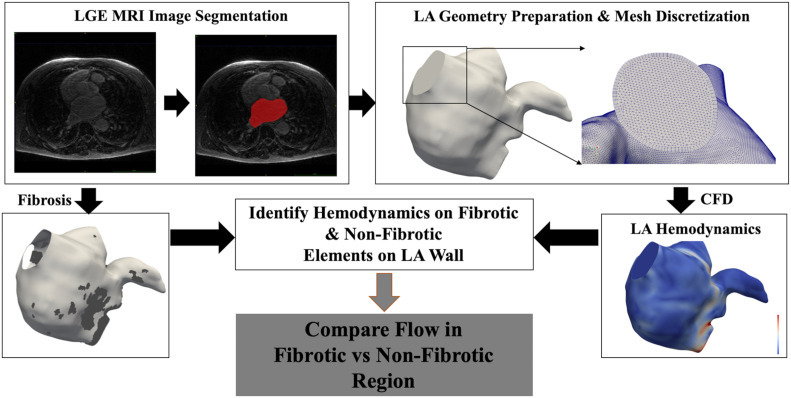
Workflow of the personalized computational fluid dynamic (CFD) simulation and comparison of hemodynamics between fibrotic and non-fibrotic regions for each case. Bottom left panel shows the left atrial (LA) model with fibrotic region highlighted in dark gray, and bottom right panel shows contour of wall shear stress (WSS) magnitude.

Subsequently, the wall of each LA hemodynamic model was divided into two interdigitated regions, fibrotic and non-fibrotic portions of the wall, each representing the group of surface elements that either have fibrosis or not. Hemodynamic values were accordingly assigned to be either on a fibrotic or a non-fibrotic portion of the wall, representing the WSS, OSI, ECAP, and RRT distributions in these two parts of the LA wall. Each LA hemodynamic model consisted of ∼20,000–50,000 data points at the LA wall, with each point either fibrotic or non-fibrotic and assigned local hemodynamic values. To compare among different cases, WSS, ECAP, and RRT values were normalized by their mean values throughout the LA model. The registration method was automated and independent of the personalized LA hemodynamic simulations. The overall workflow of the personalized hemodynamic simulations is shown in Figure 1.

To perform statistical comparison, a one-sample Kolmogorov–Smirnov test was first performed to check for normal distribution of the data. Statistical differences between the variables were then tested using the Mann–Whitney *U*-test for non-normally distributed data or Student’s *t*-test for normal data. Statistical significance was defined as *p* < 0.05. All continuous variables were expressed as mean ± standard error. Statistical analysis was performed using MATLAB (v9.7, R2019b, MathWorks, Natick, MA).

## Results

### Patient Cohort and Fibrotic Burden

A total of eight AF patients were included in this retrospective analysis: four with persistent and four with paroxysmal AF. The average volume of the LA was 158.79 ± 25.63 ml, with persistent AF patients having larger LA volumes (220.20 ± 18.61 ml) as compared to the paroxysmal AF patients (97.38 ± 14.34). The average fibrotic burden in the LA of all patients was 14.11 ± 2.05%. Among the four persistent patients, fibrosis was 12.48 ± 2.89%, while average fibrosis in the paroxysmal patients was 15.75 ± 3.09%. Case-wise values of LA volume, fibrotic burden, type of AF, and number of discretized surface points at the LA wall are provided in Table 1.

**TABLE 1:** Patient atrial fibrillation (AF) type, left atrial (LA) fibrotic burden, volume, and number of data points in each LA model for hemodynamic comparison.

Patient ID	Type of AF	LA fibrosis (%)	Volume (ml)	LA surface points
Case 1	Persistent	9.2168	190.504	40,699
Case 2	Persistent	17.768	265.038	55,573
Case 3	Persistent	16.927	236.593	49,044
Case 4	Persistent	6.0026	188.645	41,797
Case 5	Paroxysmal	13.6179	111.66	34,098
Case 6	Paroxysmal	13.4968	125.988	35,336
Case 7	Paroxysmal	11.024	92.1394	27,563
Case 8	Paroxysmal	24.8468	59.7183	19,338

### Differences in LA Hemodynamics Near Fibrotic and Non-fibrotic Portions of the Wall

Average hemodynamic comparison (Table 2) at the LA wall showed that WSS was lower and OSI was higher in fibrotic region as compared to the non-fibrotic region. However, these differences were not statistically significant, with *p*-values of 0.07 for WSS and 0.24 for OSI. However, both ECAP and RRT were significantly higher in the fibrotic region as compared to the non-fibrotic region (*p* = 0.001 and *p* = 0.002 for ECAP and RRT, respectively).

**TABLE 2:** Comparison of hemodynamics in normal and fibrotic regions averaged across eight cases.

Hemodynamic index	Non-fibrotic region	Fibrotic region	*p*-value
WSS	1.19 ± 0.13	0.95 ± 0.01	0.07
OSI	0.28 ± 0.01	0.29 ± 0.01	0.24
ECAP	0.50 ± 0.15	1.21 ± 0.10	0.001
RRT	0.49 ± 0.16	1.22 ± 0.11	0.002

**All hemodynamics values, except OSI, were normalized by their mean. WSS, wall shear stress; OSI, oscillatory shear index; ECP, endothelial cell activation potential; RRT, relative residence time.**

**FIGURE 2:**
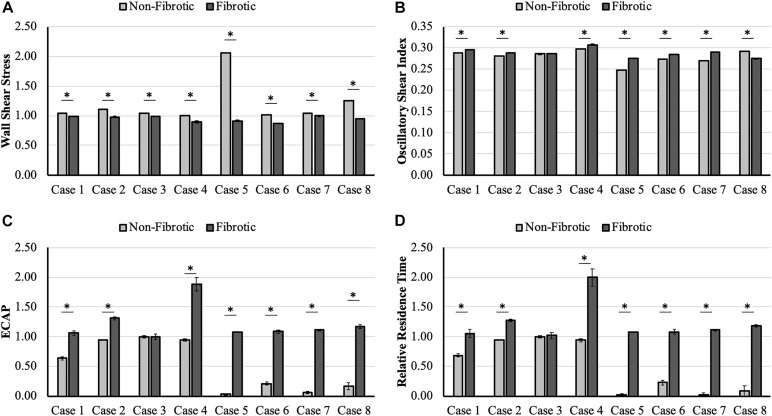
Case-wise comparison of **(A)** wall shear stress (WSS), **(B)** oscillatory shear index (OSI), **(C)** endothelial cell activation potential (ECAP), and **(D)** relative residence time (RRT) between the fibrotic and non-fibrotic left atrial (LA) wall, showing lower WSS and higher OSI, ECAP, and RRT in the fibrotic region as compared to the non-fibrotic region of the LA wall for all cases. Cases marked with * have statistically significant differences; error bars represent standard error values.

Case-wise comparison shows that for all LA hemodynamic models, WSS was consistently lower and OSI, ECAP, and RRT were consistently higher at the fibrotic portion of the wall as compared to the non-fibrotic, with differences being statistically significant for all cases, except case 3 (Figure 2). Case 3 only had significant difference in WSS (*p* < 0.001), whereas the difference in OSI (*p* = 0.05), ECAP (*p* = 0.96), and RRT (*p* = 0.71) were not statistically significant. All other cases showed strong statistically significant differences (*p* < 0.001) between all hemodynamic parameters between the fibrotic and non-fibrotic portions of the LA wall.

### Correlation of Aberrant Hemodynamics With Fibrotic Burden

**FIGURE 3:**
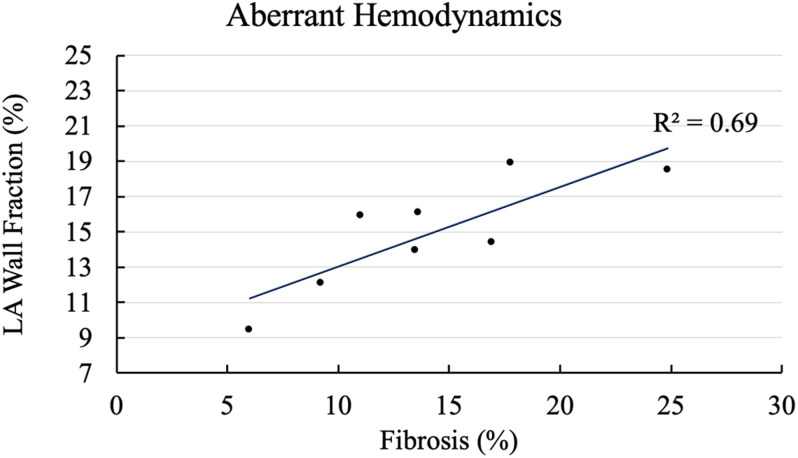
Correlation of aberrant hemodynamics with left atrial (LA) fibrotic burden. X-axis shows cases arranged with increasing fibrosis percentage, and Y-axis plots percentage of fractional LA surface area exposed to aberrant hemodynamics at LA wall.

We plotted fractional area of the LA wall exposed to aberrant hemodynamic burden captured by high ECAP as a function of fibrosis percentage for all cases in Figure 3. As shown in Figure 3, trendline shows that cases with higher fibrotic percentage tend to have higher LA area exposed to high endothelial cell activation. On the lower end, case 4 with a fibrosis of 6% had 9.4% of the total LA wall that was exposed to high ECAP. On the higher end, cases 2 and 8, which had 17.7 and 24.8% fibrosis, had 18.9 and 18.5% of the LA wall area that was exposed to high ECAP. Aberrant hemodynamic regions for all other cases lay between these extreme values; R^2^ of the correlation coefficient for the linear fit was 0.69.

### LA Hemodynamics in Representative Persistent and Paroxysmal AF Cases

**FIGURE 4:**
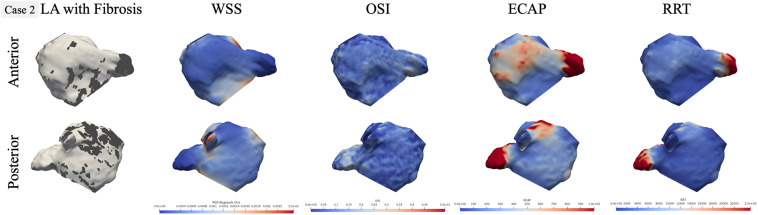
Qualitative comparison of personalized left atrial (LA) hemodynamic results with LA fibrotic burden for the representative persistent AF case 2. Dark gray region in the left panel represents the fibrosis at the LA wall. Other panels show wall shear stress (WSS), oscillatory shear index (OSI), endothelial cell activation potential (ECAP), and relative residence time (RRT) contours, respectively: blue color represents low value, and red color represents high value.

**FIGURE 5:**
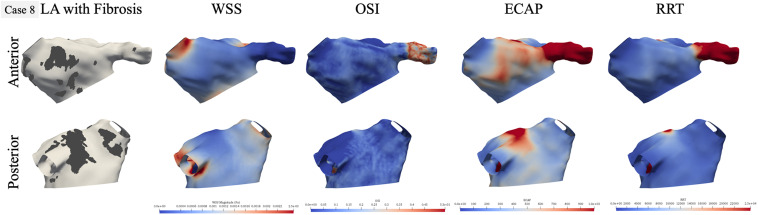
Qualitative comparison of personalized left atrial (LA) hemodynamic results with LA fibrotic burden for the representative paroxysmal atrial fibrillation (AF) case 8. Dark gray region in the left panel represents the fibrosis at the LA wall. Other panels show WSS, OSI, ECAP, and RRT contours, respectively: blue color represents low value, and red color represents high value.

To identify local hemodynamic differences at the fibrotic portion of the wall in paroxysmal and persistent AF cases, we performed qualitative comparison of hemodynamics for one representative case from each group. Cases 2 (persistent) and 8 (paroxysmal) had statistically significant differences in all hemodynamic parameters between fibrotic and non-fibrotic portions of the LA wall. For both cases, we plotted the LA geometry with the fibrotic region marked as dark gray and corresponding hemodynamic results in two views: anterior and posterior as shown in Figures 4, 5, respectively. Figure 4 shows qualitative results for the persistent AF case 2, highlighting the high amount of fibrosis in the LAA and near the MV in the anterior view and on the top of the posterior wall in the posterior view. Personalized hemodynamic results show that the fibrotic region at the LAA has locally low WSS and high ECAP and RRT in the anterior view, but OSI was not visibly different in this region. Furthermore, corresponding to the fibrosis patch at the MV, there is no aberrant hemodynamics qualitatively, likely due to the unidirectional high blood flow going out of the LA through the MV. In the posterior view, the region between the left and right inferior PVs had a large scattered patch of fibrosis, with hemodynamic values exhibiting varying patterns in this patch. There is relatively low WSS and high ECAP in the middle of left and right inferior PVs and close to the left inferior PV, but OSI and RRT do not show any differences in this region.

For the paroxysmal AF case 8, as shown in Figure 5, the fibrosis was distributed near the right superior PV in the anterior view and in two patches near the left inferior and right inferior PVs in the posterior views. In terms of hemodynamics near the fibrotic patch at the right superior PV, there is low WSS but OSI values are also low, but ECAP is higher corresponding to this region and RRT shows no difference. In the posterior view, while the fibrotic patch near the left inferior PV had low WSS, it is also the region with low OSI. The fibrosis patch near the right inferior PV does not correspond to aberrant hemodynamic, but the fibrosis patch near the left PVs does correspond to regions of high ECAP and RRT, with no differences in either WSS and OSI in this region.

## Discussion

We performed personalized CFD simulations of LA models of eight AF patients to calculate blood-flow patterns and characterize the hemodynamic differences between the fibrotic and non-fibrotic portions of the LA wall that could explain high stroke risk in AF patients with higher fibrotic burden. Current clinical management of AF patients is based on the CHA_2_DS_2_-VASc, which scores patients based on their age, sex, congestive heart failure, hypertension, prior stroke, vascular disease, and diabetes history (Chen et al., 2013; Boyle et al., 2021). However, an inherent limitation of such clinical score is the lack of use of AF patients’ atrial structural and anatomical functions in evaluating stroke risk (Yaghi and Kamel, 2017). Atria of patients with AF undergo arrhythmic remodeling and change in atrial anatomy and function (Nattel et al., 2008). Subsequently, it has been established that AF patients with higher LA fibrotic burden quantified using LGE-MRI images have higher risk of stroke (Daccarett et al., 2011). Daccarett et al., in a prospective study of 387 patients, showed that AF patients who had stroke had higher LA fibrosis as compared to those who did not (24.4 ± 12.4% vs. 16.2 ± 9.9%, *p* = 0.01). They concluded that LA fibrotic substrate was an independent predictor of stroke event and should be considered along with clinical scores to assess patients’ stroke risk stratification. However, they did not explore or hypothesize how the LA fibrosis could elicit thromboembolic event and cause stroke for patients with high LA fibrosis. In this study, we explored potential hemodynamic-based mechanisms to explain this.

The main finding of our study is that, on average, WSS was consistently lower and OSI, ECAP, and RRT were consistently higher in the fibrotic region as compared to the non-fibrotic regions for all cases, with ECAP and RRT having significant differences (Table 2). Furthermore, when we compared across all cases, we found that cases with higher fibrotic burden were exposed to larger areas of aberrant hemodynamics as shown in Figure 3, even though the correlation coefficient was not high. This suggests that (1) for each patient, fibrotic region is more susceptible to aberrant hemodynamics as compared to the non-fibrotic regions, and (2) patients with higher fibrotic burden at the LA wall have higher areas exposed to aberrant hemodynamics. These results show that fibrotic regions at the LA wall are more likely to be exposed to slower and more oscillating blood flow as compared to the non-fibrotic patches. *In vitro* studies have shown that endothelial cells activate to become proinflammatory when exposed to such low and oscillatory flow (Chiu et al., 2003; Passerini et al., 2004). We suggest that the combination of low WSS and high OSI flow is likely to activate the endothelial cell lining at the LA wall at the fibrotic region, thus attracting locally circulating platelets and leukocytes (Torisu et al., 2013; Koupenova et al., 2017). This would, in turn, activate the coagulation cascade near the endothelial cell via the venous thrombus mechanisms (Mackman, 2012), resulting in stroke-causing local clot formation. This provides a mechanistic explanation as to how the presence of fibrosis could provide sites for potential clot formation within the LA, thus increasing the risk of stroke in AF patients. However, whether these hemodynamic values are physiologically aberrant enough to activate endothelial cells and initiate the venous thrombus mechanism still remains unknown; *in vitro* studies are required to further explore this. Furthermore, even though we found significant difference in average ECAP and RRT among the fibrotic and non-fibrotic regions of the LA wall (Table 2), we did not find strong association between the increased fibrotic burden and aberrant hemodynamics (*R*^2^ = 0.69, Figure 3), suggesting that our mechanistic explanation of the activation of endothelial cells and subsequent clot formation near the fibrotic region is a hypothesis that must be tested in future studies.

Qualitative results on two representative cases show that LAA experienced aberrant hemodynamics irrespective of the fibrotic substrate. These findings provide hemodynamic evidence to further support that LAA is the most common site for clot formation within the LA (Kamp et al., 1999; Lee et al., 2014; Yaghi et al., 2015; Olivares et al., 2017; Garcia-Isla et al., 2018). In addition, we found that WSS and OSI on their own do not necessarily correspond to fibrotic regions, but higher ECAP is a better metric for visual comparison. Furthermore, not all fibrotic patches at the LA wall match perfectly with the aberrant hemodynamics, probably due to local blood-flow behaviors (Figures 4, 5). For example, in Figure 4, the region near the left inferior PV had a patch of fibrosis in the posterior view, but did not have differences in WSS or OSI, but has a presence of high ECAP. The fibrotic patch near the MV in the anterior view does not correspond to aberrant hemodynamics, which could be attributed to the local blood flow going out of the MV. Similarly, in Figure 5, the fibrotic patch near the right inferior PV for case 8 shows high WSS and low OSI, likely due to the relatively large size of the right inferior PV resulting in high amount of blood coming in to the LA. These qualitative results suggest that LA hemodynamics also depend highly on the LA and PV anatomical structure and the BCs for the blood-flow simulations. Therefore, the presence of a fibrotic patch in the LA wall does not imply the presence of a clot-prone region; the location of fibrosis at the LA wall is also an important factor. Our results indicate that blood flow near the PVs and the MV depends more on the LA structure and local flow conditions at these locations, so fibrotic substrate near these regions are less likely to have aberrant hemodynamics and therefore less clot prone. Fibrotic patches that are away from the PVs and MV could be more susceptible to result in potential clot formation sites.

CFD studies have shown that LA hemodynamics is highly dependent on incorporating the LA wall motion in the simulations (Koizumi et al., 2015; Garcia-Villalba et al., 2021). To investigate the effect of LA wall motion in our study, we performed simulations for all our models with LA wall assumed as rigid non-moving wall (Supplementary Material). As shown in Supplementary Figure 6, the rigid LA wall simulations resulted in a correlation coefficient of 0.43 between aberrant hemodynamics and LA fibrosis, as compared to 0.69 with moving LA wall simulations (Figure 3). This suggests that neglecting LA wall motion in CFD simulations might not produce accurate hemodynamics. These results are in accordance with the findings of Garcia-Villalba et al., who demonstrated that simulations with no LA wall motion results in different flow fields as compared to simulations with moving LA walls (Garcia-Villalba et al., 2021). However, our LA wall motion method is based on volume-based interpolation, which might not be representative of the physiological LA wall motion for these patients, which could be a possible reason for modest correlation coefficient. We believe that incorporating physiological LA wall motion or a mechanical LA contraction model in our simulations might result in a stronger association between LA hemodynamics and fibrosis. However, we could not incorporate physiological LA wall motion for our models due to the lack of such information in clinical imaging for these patients.

This study has some limitations. A major limitation of this study is the lack of clinical data for stroke events for these patients, so these results could not be correlated with their clinical endpoint. Second, we analyzed a small number of patients; studies on a larger cohort are needed to confirm these findings. Third, due to the lack of clinical data for LA wall motion during AF event and the flow through the PVs, we modeled the blood-flow dynamics during sinus rhythm with blood flow through the PVs and the LA wall motion interpolated from the values in the literature.

## Conclusion

We used personalized hemodynamic simulations to explore potential mechanisms that explain increased risk of stroke in AF patients with higher LA fibrotic burden. Our results show that, on average, the fibrotic portion of the LA wall had consistently lower WSS and higher OSI, ECAP, and RRT as compared to the non-fibrotic regions for all cases, with ECAP and RRT showing statistically significant differences. Additionally, cases with higher fibrotic burden have more aberrant hemodynamics as compared to cases with less fibrosis. These results suggest that the fibrotic region in the LA of AF patients might lead to aberrant hemodynamic conditions, which could lead to thrombogenesis near these areas via the venous thrombus activation, potentially causing a stroke. Amount and distribution of LA fibrotic burden could be important factors to consider while evaluating the clinical stroke risk management of AF patients.

## Data Availability Statement

The raw data supporting the conclusions of this article will be made available by the authors, without undue reservation.

## Ethics Statement

Ethical review and approval was not required for the study on human participants in accordance with the local legislation and institutional requirements. Written informed consent for participation was not required for this study in accordance with the national legislation and the institutional requirements.

## Author Contributions

NP and NT conceptualized, designed this study, analyzed the results, and drafted the manuscript. UD, TA, PP, DS, and HC acquired and transferred the clinical and imaging data. NP, RA, MS, RO’H, and RY processed the imaging data. NP performed the personalized simulations. All authors contributed to manuscript revision and approved the submitted version.

## Conflict of Interest

The authors declare that the research was conducted in the absence of any commercial or financial relationships that could be construed as a potential conflict of interest.

## Footnotes

1 www.itksnap.org

2 https://openfoam.com/

3 https://www.marcc.jhu.edu/

4 https://www.paraview.org/
