# Supplementary material for: Presence of Left Atrial Fibrosis May Contribute to Aberrant Hemodynamics and Increased Risk of Stroke in Atrial Fibrillation Patients
Source: Front Physiol. 2021 Jun 7;12:657452. doi: 10.3389/fphys.2021.657452 (PMC8215291; doi:10.3389/fphys.2021.657452)
Supplement: Supplementary file 2 [file Data_Sheet_1.DOCX]

Supplementary Material

**Intergrid Transfer Operator for Fast and Reliable Registration of Clinical Data**

We employ a projection technique to accurately and efficiently interpolate clinical data of interest among independent representations of a specific computational domain [1, 2]. Let T_h1_ and T_h2_ be two non-nested triangulations of the same domain Ω, given in this context by a left atrium (LA). T_h1_ is the mesh of the LA reconstructed from medical imaging, whereas T_h2_ is the highly refined mesh of the same LA designed for CFD simulations. We refer to h_1_ and h_2_ as the average edge lengths of the two grids. In practice, especially if h_1_»h_2_, as in this case, T_h1_ and T_h2_ boundaries do not match, since they are not overlapping polygonal surfaces made of piecewise linear elements. Thus, some vertices of T_h1_ can lie outside T_h2_, as illustrated in Supplementary Figure 1. Given a scalar or vector field f(**x**), which coincides with a certain clinical data (e.g. intensities) distributed in space over T_h1_, our aim is to accurately and efficiently recover this field on mesh T_h2_. We proceed in two different ways whether vertices of T_h2_ lie inside or outside T_h1_.

In particular:

- for the internal vertices - denoted as **x**^i^_h2_, i = 0, 1, 2, … - we find the element K^i^∈T_h1_, such that **x**^i^_h2_∈K^i^, and we evaluate f(**x**^i^_h2_) by exploiting its Finite Element expansion on K^i^;
- for each external point - denoted as **x**^j^_h2_, j = 0, 1, 2, … - more sub-steps are necessary:
  1. we find the closest element K^j^∈T_h1_ from the external point **x**^i^_h2_;
  2. on K^j^, we find the closest point **x**^i^_h1_ for **x**^j^_h2_;
  3. we compute f(**x**^i^_h1_) and we project the result on the external point **x**^j^_h2._

Our interpolant is developed in the framework of VMTK ([www.vmtk.org](http://www.vmtk.org)) [3], which is based on the VTK library [4]. We used the recently proposed tools for cardiac mesh generation and preprocessing [1]. These tools contain filtering utilities to locate all internal points, which are the majority, in a really fast manner. Moreover, it performs closest points interpolation efficiently, leading to an accurate procedure. For further mathematical details about this technique, we refer to [1, 2].

In Supplementary Figure 2 we show the triangulations T_h1_ (left) and T_h2_ (right) of a patient-specific LA. The two meshes present a really different space resolution (i.e. h_1_»h_2_). Finally, in Supplementary Figure 3, we depict the results of our interpolation method on the same LA. Precisely, we transfer intensity values from T_h1_ (left) to T_h2_ (right).

**Details of Computational Fluid Dynamics Methodology**

1. *Inlet velocity waveform at the pulmonary veins (PVs):* We identified two peaks during a cardiac cycle of the pulmonary venous flow: systolic and diastolic. Then, we assessed the average magnitude values of peak systolic velocity and peak diastolic velocity and the wave duration from a previous study based on doppler measurements from the report by Gentile et al.[5] We then interpolated and normalized these average values to obtain a generic velocity waveform that represents the PV flow as shown in Supplementary Figure 4. The duration of the waveform is 0.92 seconds, representing the cycle length of 1 cardiac cycle. Then before performing flow-simulations, for each case, the waveform was scaled according to the area of each of the 4 PVs and used as the inlet boundary condition for the simulations. We then multiplied this velocity profile by the unit normal vector of each PV to ensure normal flow entering from the PVs for each case.
2. *CFD Equations and discretization schemes:* The flow-governing Navier-Stokes’ equations were solved for each case:

$\nabla.v=0$ (1)

$\frac{\partial v}{\partial t}+v.\nabla v=-\frac{1}{\rho}\nabla p+\frac{\mu}{\rho}\nabla^{2}v$ (2)

where *v* is velocity, *p* is pressure, $\mu$ is viscosity and $\rho$ is density. We used 1^st^ order implicit temporal Euler discretization to discretize the time and 2^nd^ order spatial linear discretization for space. Each of the 4 PVs served as the inlet for the blood-flow with adjusted normal velocities prescribed as mentioned in the previous section. Zero pressure boundary conditions were prescribed at the mitral valve that served as the outlet for the blood-flow.

1. *Left atrial wall motion:* To model the LA wall motion, we first identified average left atrial volume variation in AF patients based on Habibi et al.[6] Three regions were identified during the LA wall motion: reservoir, conduit and pump. The temporal occurrence of peak LA volume at the end of reservoir and conduit were normalized on a temporal cycle length of 0.92 seconds. Then average LA volume values were identified at each of these peaks and interpolated over the whole cycle. The final interpolated LA volume change that we implemented is shown in Supplementary Figure 5. For each case, we assumed the initial LA segmentation as the LA volume at t=0, and then LA volume change during the cardiac cycle were prescribed using the waveform shown in Supplementary Figure. 5. To obtain the desired LA volume change, a uniform constrained motion was applied on each element of the LA wall surface, and the subsequent volume mesh expanded or contracted accordingly. This constrained motion of the LA wall ensured that the computational mesh did not change over time; each volumetric cell just changed its shape based on the prescribed motion. Due to the volume mesh deformation, the subsequent velocity interpolations between time-steps were solved by using the Laplace equation in the pimpleFoam solver (<https://openfoam.com/>), which interpolates velocity and pressure values based on the dynamically moving mesh.[7]
2. *Difference between hemodynamics in LA models with and without wall motion:* To compare the sensitivity of our results to LA wall, we performed CFD simulation with LA wall assumed as rigid for each model. We then calculated the aberrant hemodynamics area for each case and plotted it against LA fibrosis percentage. Results of our analysis are shown in Supplementary Figure 6, which shows a correlation coefficient of 0.43. This clearly demonstrates that CFD simulations that incorporate LA wall motion provide better correlation of LA fibrosis with hemodynamics, and might provide more accurate LA hemodynamics as compared to those with rigid LA wall assumption.

CFD results for the LA hemodynamic model for a representative (Case 8) are provided in the Supplementary Video that shows the LA wall motion and the wall shear stress magnitude contour on the LA surface in the anterior view throughout one cardiac cycle during sinus rhythm.

**Equations for Hemodynamic Parameter Calculations**

Time-averaged wall shear stress (WSS) was computed as the average of the WSS vector over a cardiac cycle during sinus rhythm based on the following equation:

$WSS=\frac{1}{T}\int_{0}^{T} \left| {wss}_{i} \right| dt$ (3)

Oscillatory shear index (OSI) was computed as the directional change in the WSS vector over a cardiac cycle in sinus rhythm using the following equation:

$OSI=\frac{1}{2}\left\{ 1-\frac{\left| \int_{0}^{T} {wss}_{i} dt \right|}{\int_{0}^{T} \left| {wss}_{i} \right| dt} \right\}$ (4)

Endothelial cell activation potential was calculated as the ratio of the time-averaged WSS and OSI for one cardiac cycle using the following equation:[8]

$ECAP=\frac{OSI}{WSS}$ (5)

Relative residence time was also computed using the WSS and OSI values by using the following equation:[9]

$RRT=\frac{1}{\left( 1-2*OSI \right)*WSS}$ (6)

T represents the cycle length and *i* indicate the WSS vector in Einstein notation.

**Supplementary Figures**


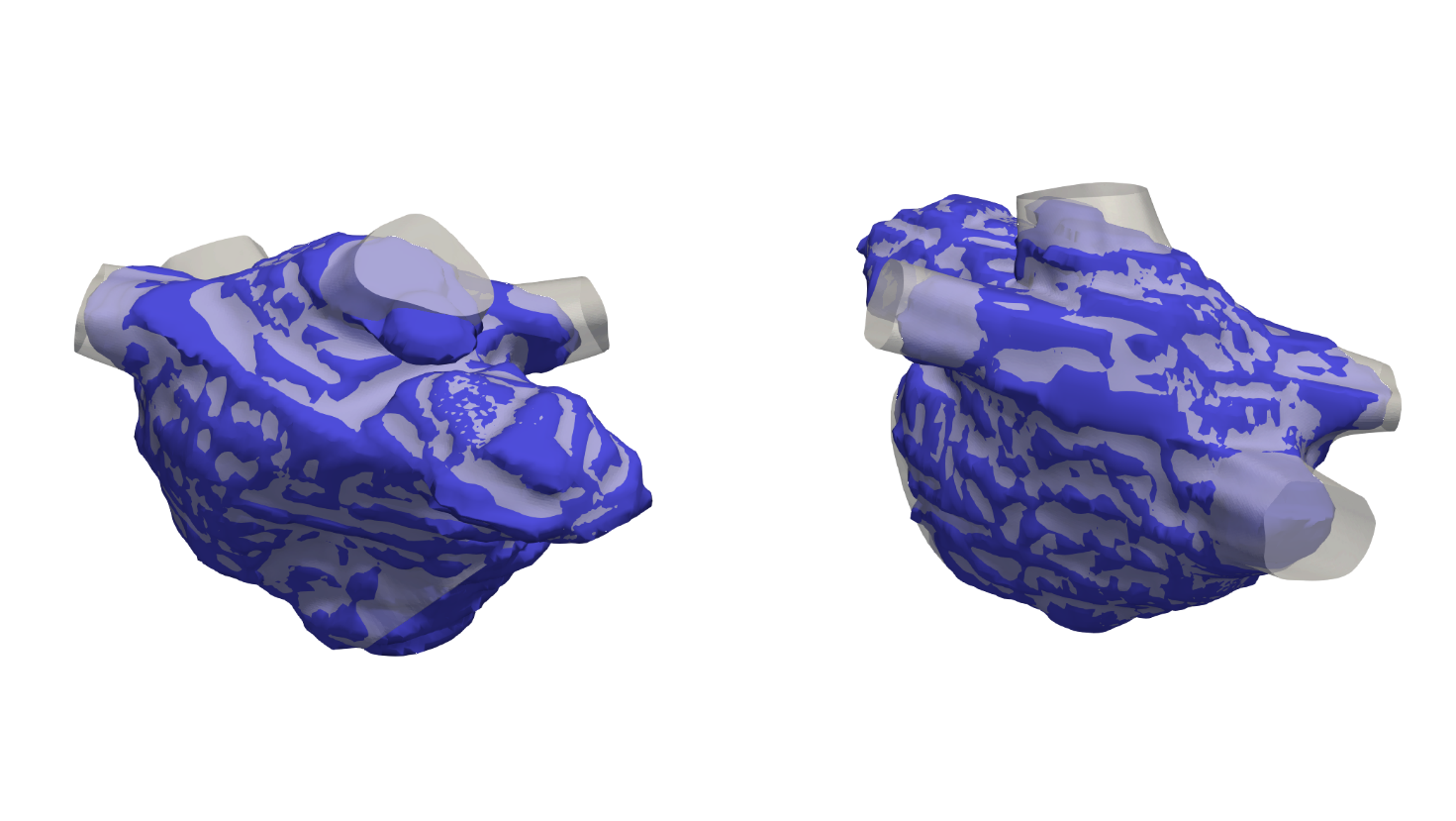


Supplementary Figure 1. Case 2: geometrical differences between the LA reconstructed from medical imaging (light blue) and the one used for CFD simulations (white). We depict a front view (left) and a rear view (right) of the LA. We stress that the two epicardial surfaces are not perfectly matching.


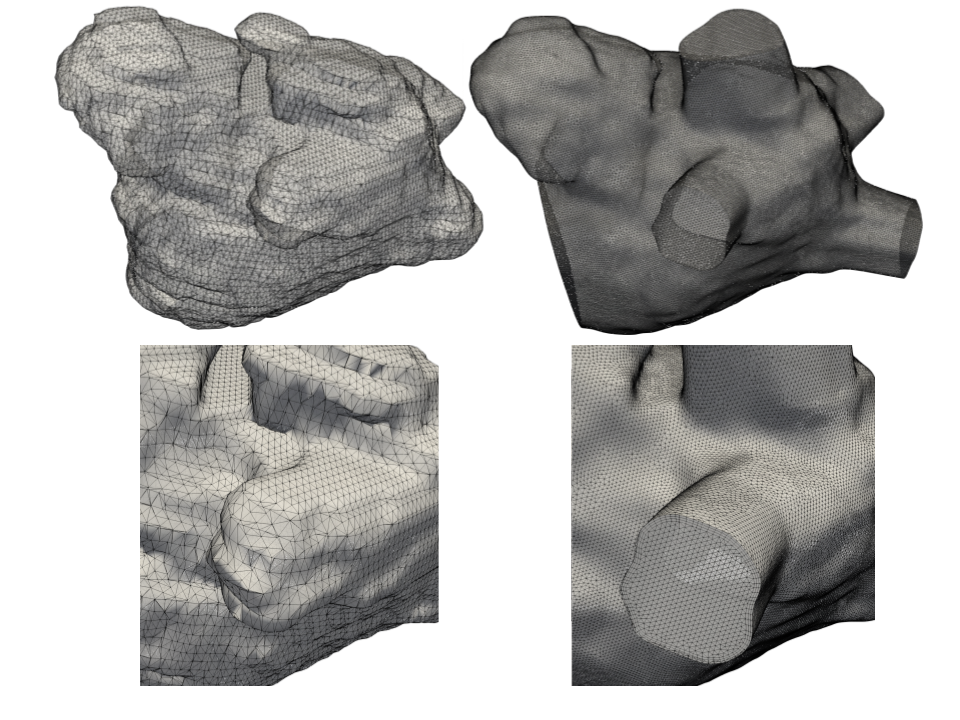


Supplementary Figure 2. Case 2: independent tetrahedral meshes, either coming from clinical data (left) or used in CFD simulations (right). We also propose a close-up on a pulmonary vein to underline the differences in the distribution of the Finite Elements.


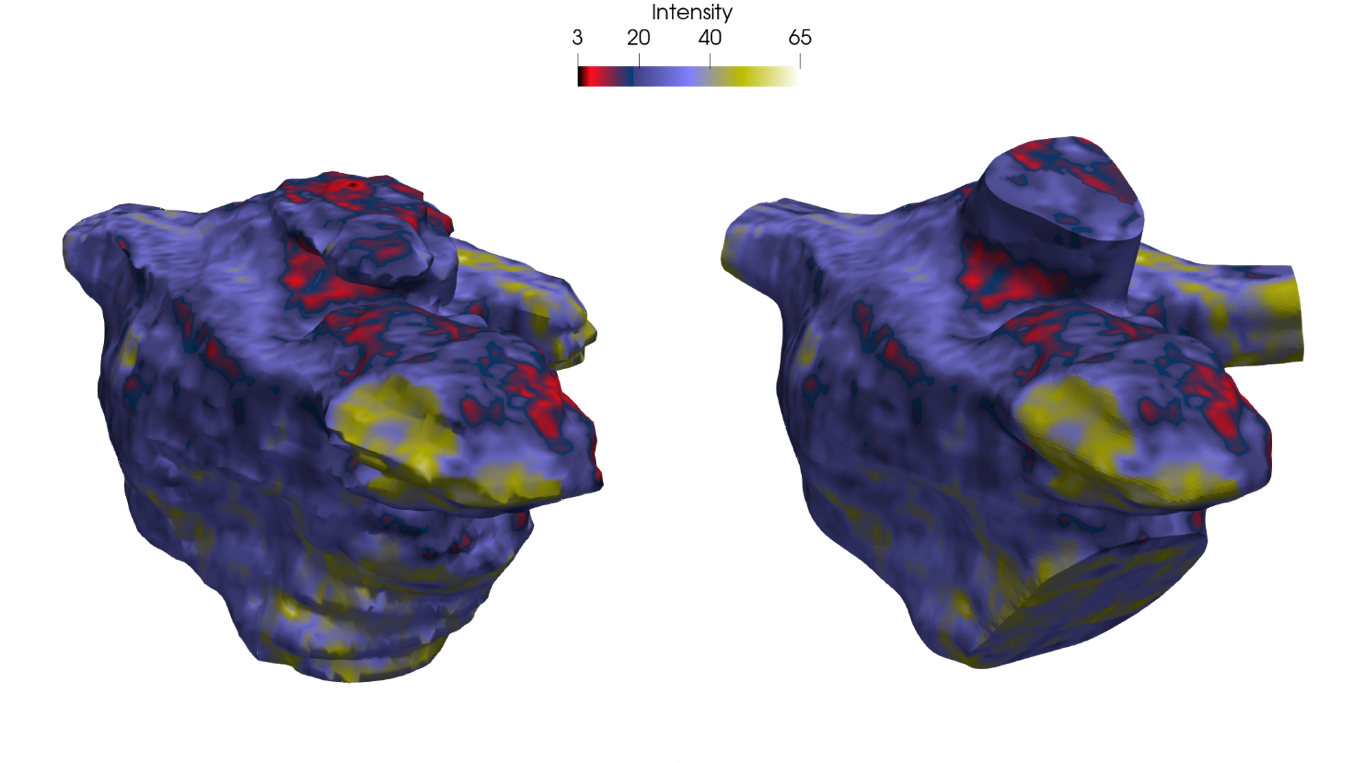


Supplementary Figure 3. Case 2: comparison between the intensity scalar field retrieved from medical imaging (left) and the interpolated one ready for CFD simulations (right). We highlight that the geometrical differences entail minor effects on the final result.


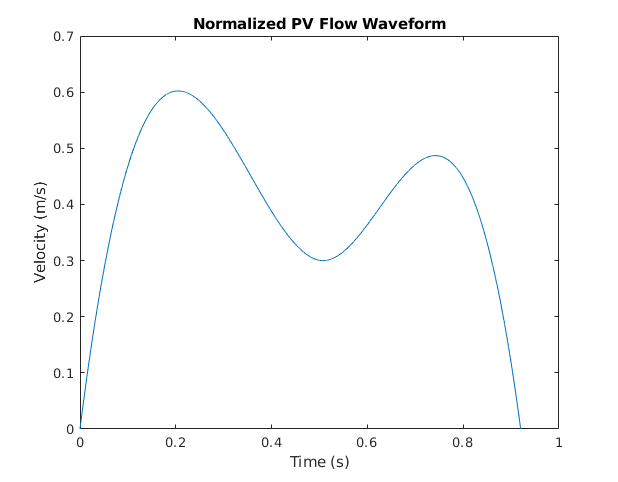


Supplementary Figure 4. Generic inlet velocity flow waveform at the pulmonary veins.


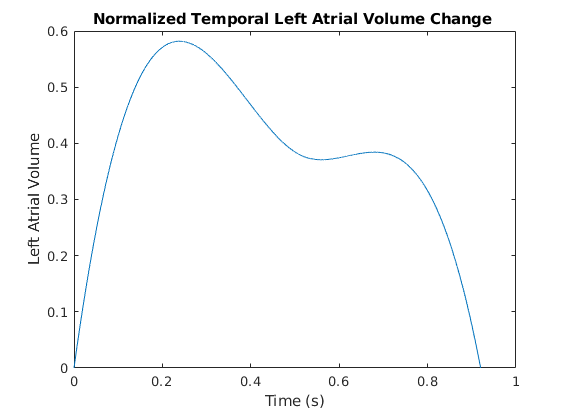


Supplementary Figure 5. Normalized LA volume change during a cardiac cycle.

**
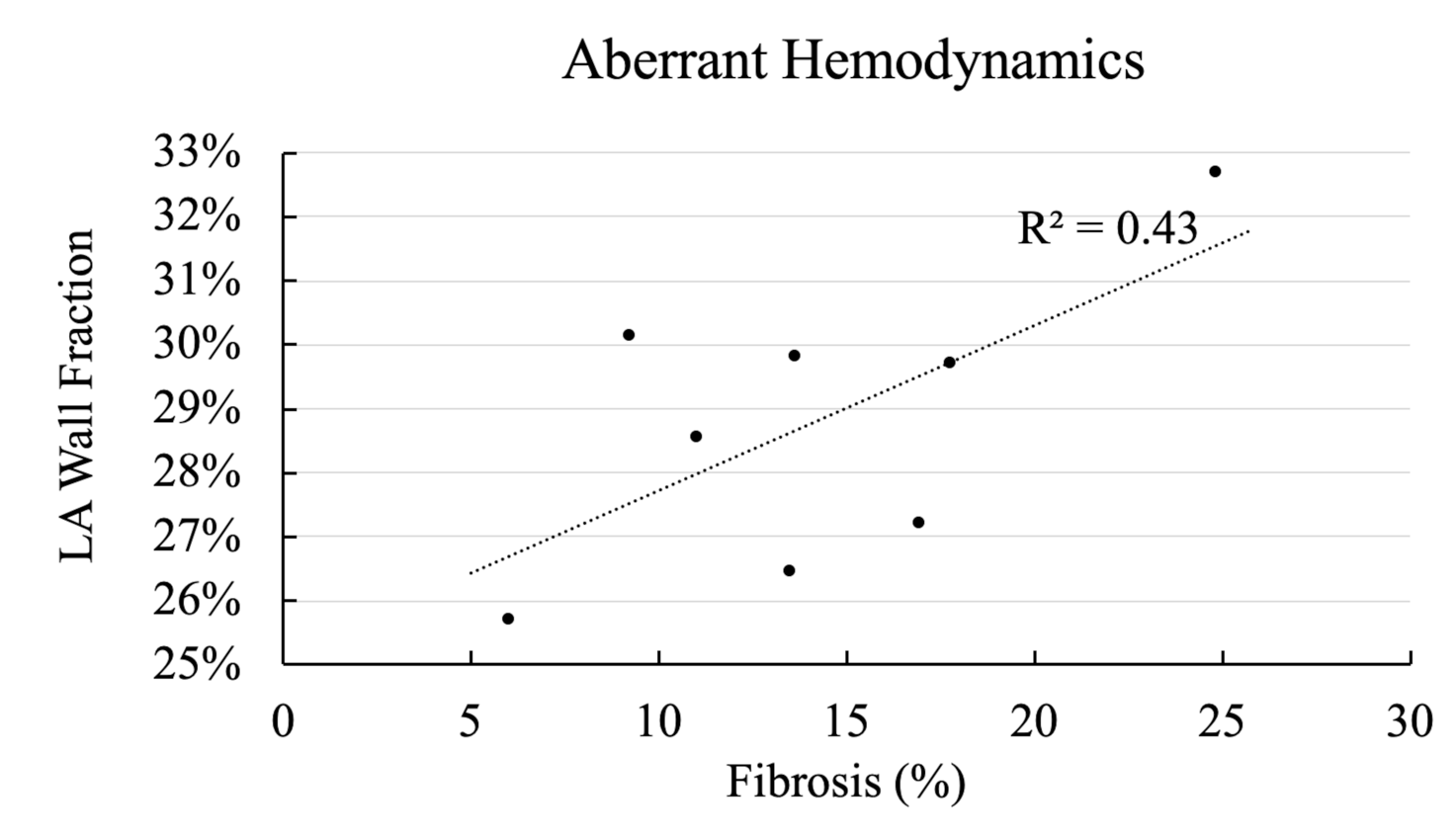
**

Supplementary Figure 6. Correlation of aberrant hemodynamics with LA fibrotic burden for simulations with rigid LA wall simulations. X-axis shows cases arranged with increasing fibrosis percentage and Y-axis plots percentage of fractional LA surface area exposed to aberrant hemodynamics at LA wall.

**Supplementary References**

[1]  M. Fedele and A. Quarteroni, Polygonal surface processing and mesh generation tools for the numerical simulation of the cardiac function. *Int J Numer Meth Biomed Engng* (2021). Accepted Author Manuscript e3435.

[2] F. Regazzoni, M. Salvador, P.C. Africa, M. Fedele, L. Dede', and A. Quarteroni. A cardiac electromechanics model coupled with a lumped parameters model for closed-loop blood circulation. Part II: numerical approximation. *arXiv preprint arXiv:2011.15051* (2020).

[3] L. Antiga, M. Piccinelli, L. Botti, et al. An image-based modeling framework for patient-specific computational hemodynamics. *Med Biol Eng Comput 46, 1097* (2008)

[4] W. Schroeder, K. Martin, and W. Lorensen. The Visualization Toolkit, An Object-Oriented Approach to 3D Graphics. *Kitware* (2006).

[5] Gentile, F., A. Mantero, A. Lippolis, M. Ornaghi, M. Azzollini, P. Barbier, L. Beretta, F. Casazza, R. Corno, F. Faletra, E. Giagnoni, C. Gualtierotti, S. Lombroso, R. Mattioli, A. Morabito, M. Pepi, S. Todd, and A. Pezzano. 1997. "Pulmonary venous flow velocity patterns in 143 normal subjects aged 20 to 80 years old. An echo 2D colour Doppler cooperative study." Eur Heart J 18 (1):148-64.

[6] Habibi M, Lima JA, Khurram IM, Zimmerman SL, Zipunnikov V, Fukumoto K, Spragg D, Ashikaga H, Rickard J, Marine JE, Calkins H. Association of left atrial function and left atrial enhancement in patients with atrial fibrillation: cardiac magnetic resonance study. Circulation: Cardiovascular Imaging. 2015 Feb;8(2):e002769.

[7] Otani T, Al-Issa A, Pourmorteza A, McVeigh ER, Wada S, Ashikaga H. A computational framework for personalized blood flow analysis in the human left atrium. Annals of biomedical engineering. 2016 Nov;44(11):3284-94.

[8] Di Achille P, Tellides G, Figueroa CA, Humphrey JD. A haemodynamic predictor of intraluminal thrombus formation in abdominal aortic aneurysms. Proceedings of the Royal Society A: Mathematical, Physical and Engineering Sciences. 2014 Dec 8;470(2172):20140163.

[9] Lee SW, Antiga L, Steinman DA. Correlations among indicators of disturbed flow at the normal carotid bifurcation. Journal of biomechanical engineering. 2009 Jun 1;131(6).
